# Erratum to Prevalence and clinical significance of pathogenic germline *BRCA1/2* mutations in Chinese non-small cell lung cancer patients

**DOI:** 10.20892/j.issn.2095-3941.2020.0029

**Published:** 2020-05-15

**Authors:** Xingsheng Hu, Dongyong Yang, Yalun Li, Li Li, Yan Wang, Peng Chen, Song Xu, Xingxiang Pu, Wei Zhu, Pengbo Deng, Junyi Ye, Hanhan Zhang, Analyn Lizaso, Hao Liu, Xinru Mao, Hai Huang, Qian Chu, Chengping Hu

**Affiliations:** ^1^Department of Medical Oncology, National Cancer Center/National Clinical Research Center for Cancer/Cancer Hospital, Chinese Academy of Medical Sciences and Peking Union Medical College, Beijing 100021, China; ^2^Department of Pulmonary Medicine, Second Affiliated Hospital of Fujian Medical University, Quanzhou 362100, China; ^3^Department of Respiratory Medicine, West China Hospital, Sichuan University, Chengdu 610041, China; ^4^Department of Respiratory Medicine, Daping Hospital, Chongqing 400042, China; ^5^Department of Respiratory Medicine, Xinqiao Hospital, Army Medical University, Chongqing 400037, China; ^6^Department of Medical Oncology, Tianjin Medical University Cancer Institute and Hospital, National Clinical Research Center for Cancer; Key Laboratory of Cancer Prevention and Therapy, Tianjin; Tianjin’s Clinical Research Center for Cancer, Tianjin 300060, China; ^7^Department of Thoracic Oncology, Tianjin Medical University General Hospital, Tianjin 300052, China; ^8^Department of Medical Oncology, Hunan Cancer Hospital, Changsha 410006, China; ^9^Department of Pathology, Xiangya Hospital, Central South University, Changsha 410008, China; ^10^Department of Respiratory Medicine, Xiangya Hospital, Central South University, Changsha 410008, China; ^11^Burning Rock Biotech, Guangzhou 510300, China; ^12^Department of Oncology, Tongji Hospital of Tongji Medical College, Huazhong University of Science and Technology, Wuhan 430022, China

In the published article^1^, the affiliation for the first author, Xingsheng Hu, is “Department of Medical Oncology, Cancer Hospital, Chinese Academy of Medical Sciences”, we would like to update it to “Department of Medical Oncology, National Cancer Center/National Clinical Research Center for Cancer/Cancer Hospital, Chinese Academy of Medical Sciences and Peking Union Medical College, Beijing, 100021, China”. We apologize for the errors and for any confusion it may have caused.

## References

[1] Hu X, Yang D, Li Y, Li L, Wang Y, Chen P (2019). Prevalence and clinical significance of pathogenic germline *BRCA1*/*2* mutations in Chinese non-small cell lung cancer patients.. Cancer Biol Med..

